# Optimized Simultaneous Assessment of Subcortical and Cortical Auditory Responses Through a Frequency‐Tagged Roving Paradigm

**DOI:** 10.1111/ejn.70337

**Published:** 2025-11-29

**Authors:** Xiaoyu Wang, Loretta Norton, Teneille E. Gofton, Derek B. Debicki, Marat Slessarev, Adrian M. Owen

**Affiliations:** ^1^ Western Institute of Neuroscience Western University London Canada; ^2^ Department of Physiology and Pharmacology Western University London Canada; ^3^ Department of Psychology, King's University College Western University London Canada; ^4^ Department of Clinical Neurological Sciences, Schulich School of Medicine and Dentistry Western University London Canada; ^5^ Department of Medicine, Schulich School of Medicine and Dentistry Western University London Canada; ^6^ Department of Psychology Western University London Canada

**Keywords:** auditory evoked potential (AEP), auditory steady‐state response (ASSR), event‐related potentials (ERP), frequency‐following response (FFR), mismatch negativity (MMN)

## Abstract

Assessment of auditory‐evoked responses across multiple stages of the ascending auditory pathway provides complementary insights into neural integrity for research and clinical contexts. However, traditional approaches, constrained by conflicting optimal parameters, require separate sessions for different response types, limiting efficiency and preventing simultaneous multi‐level assessment, while evidence of individual‐level sensitivity and reliability remains limited. We aimed to develop and validate a paradigm enabling concurrent, single‐subject assessment of frequency‐following responses (FFRs), auditory steady‐state responses (ASSRs), and event‐related potentials (ERPs) spanning subcortical to cortical levels. Two amplitude‐modulated tones (carriers at 220/440 Hz, modulated at 40/80 Hz) were presented in a roving sequence so that each tone served as both standard and deviant, and EEG was recorded using a two‐electrode montage (Fz, Cz) EEG setup. In 32 healthy participants, the paradigm achieved 100% sensitivity for high‐frequency FFRs and gamma‐band ASSRs, confirmed by permutation‐based spectral analysis. Machine‐learning classification distinguished stimulus conditions from resting state based on N1 and sustained negativity in all participants (32/32), confirming robust single‐subject detection of obligatory cortical responses. Directional asymmetry was observed in transition responses: ascending frequency transitions predominantly elicited enhanced N1–P2‐like responses (32/32), whereas descending transitions evoked mismatch negativity–like (MMN‐like) responses in 30/32 participants. Recording‐duration analysis showed that overall detection sensitivity across response components reached 0.91 after 27 min of recording. Collectively, these findings indicate that the frequency‐tagged roving paradigm provides a framework for characterizing auditory processing across hierarchical levels within a single session, supporting potential use in future experimental and translational studies.

## Graphical abstract

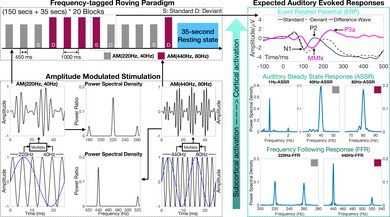

**Abbreviations**
AEP: auditory‐evoked potential
AM: amplitude‐modulated
ASSR: auditory steady‐state response
DFT: discrete Fourier transform
DOC: disorders of consciousness
ERP: event‐related potential
FFR: frequency‐following response
ML: machine learning
MMN: mismatch negativity
PSD: power spectral density
RS: resting state
SNR: signal‐to‐noise ratio
SOA: stimulus onset asynchrony
SVM: support vector machine
TFCE: threshold‐free cluster enhancement

## 1 Introduction

The auditory system dynamically transforms acoustic signals into meaningful neural representations along the ascending auditory pathway, extending from the cochlea through brainstem nuclei, midbrain, thalamus, to the auditory cortex (Peterson et al. 2024; Schnupp 2011). The integrity of this hierarchical system supports normal auditory function, and disruption at different levels can manifest as distinct functional impairments. Consequently, precise characterization of processing deficits across these stages is important for accurate assessment and targeted intervention. This need is particularly evident in behaviorally nonresponsive populations, ranging from acute coma to chronic disorders of consciousness (DOC), where characterization of preserved auditory function provides information relevant to diagnostic and prognostic evaluation (Comanducci et al. 2020; Norton et al. 2023). In such individuals, objective electrophysiological measures can provide valuable indicators of residual neural activity without requiring active participation (Krizman and Kraus 2019; Picton et al. 2000).

Auditory‐evoked potentials (AEPs), recorded as electrical brain activity through scalp electrodes, capture neural processing with millisecond precision across multiple stages of the auditory pathway (Picton 2010). Sustained AEPs represent a distinct category of responses, characterized by frequency‐tagged neural activity arising from synchronous population‐wide phase‐locking to periodic features throughout stimulus presentation (Skoe and Kraus 2010). Within this category, the frequency‐following response (FFR), arising primarily from brainstem generators including the inferior colliculus, is thought to reflect the subcortical encoding of temporal periodicities ranging approximately from 100 to 1500 Hz, typically representing fundamental frequency and harmonics of simple sinusoidal tones, complex vowels, and musical notes (Aiken and Picton 2008; Lee et al. 2009; Slugocki et al. 2017). Similarly, the auditory steady‐state response (ASSR) represents another form of neural entrainment to periodic auditory stimulation, primarily studied at modulation frequencies in the gamma band (30–80 Hz) (Korczak et al. 2012; Picton et al. 2003). These responses are associated with at least partially distinct neural generators depending on the modulation frequency, with the 40‐Hz ASSR thought to predominantly reflect cortical/thalamocortical circuits, and the 80‐Hz ASSR showing stronger contributions from subcortical structures (Farahani et al. 2021; Herdman et al. 2002). These ASSRs can be elicited through either amplitude‐modulated (AM) tones with carrier‐specific modulation frequencies (Becher et al. 2015; Farahani et al. 2021) or brief click trains presented at rates matching the desired response frequency (Neklyudova et al. 2024; Seymour et al. 2020).

Complementing these sustained responses, transient AEPs are characterized by voltage deflections time‐locked to discrete acoustic events such as sound onset and offset (Skoe and Kraus 2010). Cortical event‐related potentials (ERPs) represent a subset of these responses that reflect the sequential stages of sound processing through distinct components, progressing from early sensory encoding to later cognitive processing (Luck 2014). These components are typically studied using auditory oddball paradigms, where infrequent deviant sounds are pseudorandomly presented within a stream of repetitive standard stimuli. In a passive oddball paradigm, where participants are instructed to ignore the sounds, each stimulus elicits an obligatory P1‐N1‐P2 complex (50–200 ms), largely generated within auditory‐cortical areas, marking the initial cortical registration and feature extraction (Näätänen and Picton 1987; Tomé et al. 2015). When deviants violate the established pattern of standard sounds, a mismatch negativity (MMN, 100–250 ms) is observed over temporal and frontal regions, reflecting automatic detection of acoustic pattern violations (Näätänen et al. 1978; Näätänen et al. 2019). This deviance detection can trigger a subsequent P3a component (250–350 ms) in frontotemporal networks, reflecting involuntary reorienting of attention to the detected change (Polich 2007).

Sustained and transient AEPs complement each other in characterizing different aspects of auditory processing across hierarchical levels, spanning from early subcortical encoding to later cortical pattern detection. These responses, however, differ markedly in their temporal characteristics and recording requirements and are therefore not usually recorded concurrently. Specifically, sustained frequency‐tagged responses tolerate high stimulation rates (interstimulus interval, ISI 10–50 ms) and are well‐suited for rapid presentation paradigms but require averaging over thousands of trials to achieve adequate signal‐to‐noise ratios (SNR) due to their inherently small amplitudes (typically in nanovolts) (Bidelman 2015a; Krizman and Kraus 2019). In contrast, transient ERPs require longer interstimulus intervals (typically > 500 ms) to ensure full neural recovery and achieve reliable responses with several hundred trials (Luck 2014). For example, N1 amplitude decreases as ISI shortens, quantitatively illustrating why rapid stimulus presentation is challenging for cortical response recording (Francisco et al. 2020). Due to these differing requirements, these responses are typically recorded in separate sessions (Bidelman et al. 2014; Wible et al. 2005). However, such separation can be particularly problematic in behaviorally nonresponsive populations, who often show fluctuating arousal levels that complicate data acquisition and interpretation (Armanfard et al. 2019; Edlow et al. 2021). Therefore, developing methods that enable simultaneous recording may provide a more practical and reliable means of evaluating auditory hierarchy.

Given these challenges, recent methodological advances have demonstrated the feasibility of simultaneously recording subcortical and cortical AEPs through innovative approaches. Early attempts primarily combined brainstem FFR with early cortical P1‐N1‐P2 complex using speech syllables (Musacchia et al. 2008) or iterated rippled noise stimuli (Krishnan et al. 2012), each repeated thousands of times. This concurrent recording approach was further applied to investigate speech categorization (Ou and Yu 2022) and spectrotemporal processing characteristics (Calcus et al. 2022). To improve recording efficiency, Bidelman (2015b) developed a clustered stimulus paradigm, in which brief trains of 14 rapid speech stimuli with a 50‐ms ISI were presented to evoke the FFR, interspersed with longer 1500‐ms intervals to elicit ERPs. Building upon these foundations, later studies expanded the paradigm by incorporating multiple stimulus conditions to enable assessment of automatic change detection through MMN (Cheng and Zhao 2024; Font‐Alaminos et al. 2021). Slugocki et al. (2017) further employed amplitude‐modulated (AM) stimuli to extend the frequency span of simultaneous recording, eliciting both subcortical and cortical responses (e.g., 500‐Hz FFR, N1–P2, MMN) while incorporating additional frequency‐tagged information via 37 and 81‐Hz ASSRs.

Despite these methodological advances, several challenges remain in optimizing the elicitation and detection of concurrent auditory responses at the individual level, particularly for applications requiring stable recordings in behaviorally nonresponsive populations. For sustained components, while Slugocki et al. (2017) additionally elicited both low‐ and high‐gamma ASSRs using multiplexed AM stimulations, emerging evidence suggests that alternative paradigms employing single AM stimuli can yield better SNRs (Sonck et al. 2025). Further, these paradigms rely on extensive repetition of standard stimuli to define auditory regularities, which can lead to gradual decreases in cortical response amplitude across trials (Francisco et al. 2020; Mill et al. 2011). Roving paradigms provide a dynamic framework, as each tone probabilistically serves as both standard and deviant within the sequence, enabling dynamic updating of auditory regularities (Garrido et al. 2008; Merchie and Gomot 2023). Additionally, these paradigms often require extended continuous recordings to collect sufficient trials, which can be challenging to maintain in behaviorally nonresponsive individuals with fluctuating arousal states (Edlow et al. 2021). Such state‐dependent fluctuations can compromise response reliability across time windows and complicate comparisons across different processing levels. As Armanfard et al. (2019) demonstrated, prolonged averaging across extended recording periods can substantially attenuate ERPs in nonresponsive individuals, whereas shorter analysis windows (2–3 min) may reveal transient components otherwise undetectable.

To address these limitations, we designed and evaluated a frequency‐tagged roving paradigm incorporating several key innovations. First, to improve signal quality for sustained AEPs, we employed single AM stimuli by pairing carrier frequencies (220 Hz/440 Hz) with distinct modulation rates (40 Hz/80 Hz), enabling simultaneous assessment of FFRs and ASSRs. Second, we employed a roving sequence, in which each tone probabilistically transitions between standard and deviant conditions, enabling continuous updating of auditory regularities and maintaining response stability over repeated stimulation. Third, we implemented a block design with 2.5‐min active stimulus periods separated by 35‐s silent intervals, enabling short‐window analyses of transient neural dynamics and improving recording stability under variable arousal or vigilance conditions. This design enables systematic characterization of auditory processing from subcortical encoding to cortical change detection, while controlling for stimulus‐specific effects. We validated this approach in healthy participants, showing that it reliably elicited multiple AEP components with high single‐subject detection rates. This work provides a methodological basis for multi‐level assessment of auditory processing, with potential applications in contexts requiring efficient and reliable measurement of auditory responsiveness.

## 2 Material and Methods

### 2.1 Participants

A total of 32 adult participants (mean age 26.88 ± 6.73 years; 13 males) were recruited from Western University. All participants self‐reported normal hearing at the time of the study and had no history of neurological or psychiatric illnesses. Participants were paid for their involvement and gave written informed consent prior to the study. The research protocol was approved by Western University's Health Sciences Research Ethics Board (project identification number: 114967).

### 2.2 Stimuli

**FIGURE 1:**
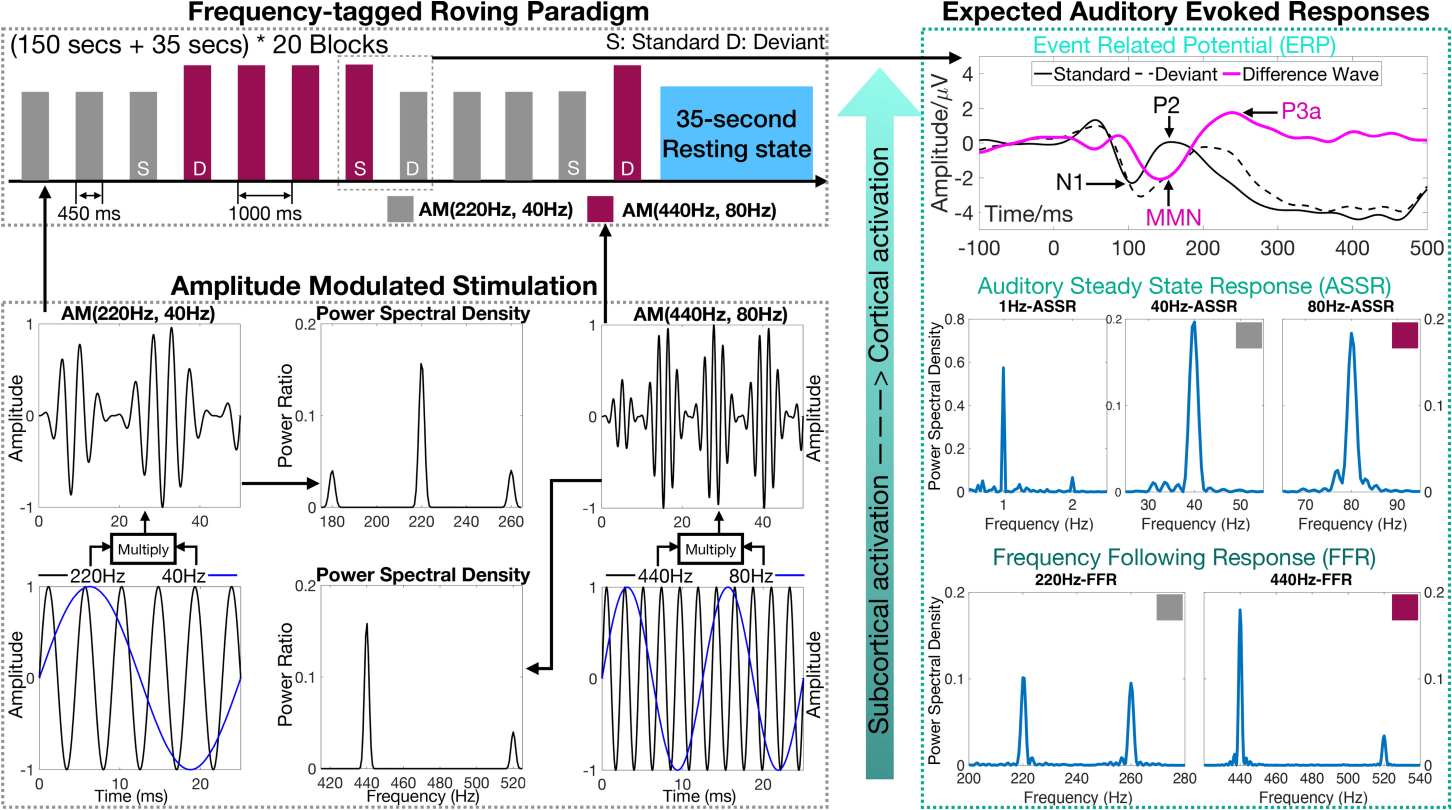
Schematic of the frequency‐tagged roving paradigm and associated auditory response domains. Left, top: The roving paradigm structure consisted of 20 stimulus blocks, each followed by a 35‐s resting interval. Each block contained 150 trials of AM tones, with each trial comprising a 450‐ and a 550‐ms interstimulus interval. Within each block, one of the two AM tones (AM (220 Hz, 40 Hz) or AM (440 Hz, 80 Hz)) was repeated pseudorandomly 4–6 times before switching to the other AM tone. The first stimulus of each new tone‐train (except the initial train) was designated as the deviant (D), and the last stimulus of the preceding train was designated as the standard (S). Left, bottom: Example waveforms and power spectra of the two AM tones. Right: Expected auditory responses spanning subcortical and cortical domains, including ERPs (top), ASSRs (middle), and FFRs (bottom).

Auditory responses were elicited using single AM tones, defined according to the following formula:

$$y\left(t\right)=\left(1+\sin\left(2\pi f_{m}t\right)\right)\times \sin\left(2\pi f_{c}t\right)$$

where $y\left(t\right)$ is the AM signal at time t, $f_{c}$ is the carrier frequency, and $f_{m}$ is the modulation frequency with 100% modulation depth. Using this formula, two distinct AM tones were employed: AM (220 Hz, 40 Hz) and AM (440 Hz, 80 Hz), with each tone specified by its carrier and modulation frequencies, respectively, as shown in Figure 1. These specific frequencies were selected based on prior evidence of optimal phase‐locking in the auditory system (Tichko and Skoe 2017). Each stimulus lasted 450 ms with 10‐ms rise/fall times to minimize spectral splatter. Stimuli were generated using Python (NumPy library) at 48‐kHz sampling rate.

During the experiment, participants were seated in a comfortable chair within a standard EEG booth. Auditory stimuli were delivered binaurally through insert earphones (ER‐3A, Etymotic Research, IL, USA) at a comfortable listening level. Stimulus presentation was controlled via Psychopy software (Peirce 2009) and Cedrus StimTracker (Cedrus Corporation, San Pedro, CA, USA) using fixed polarity. To maintain arousal while ensuring passive listening conditions, participants were instructed to read a book or watch a silent movie of their choice while ignoring the auditory stimuli, consistent with standard passive auditory protocols (Picton et al. 2000; Näätänen et al. 2007). No behavioral responses were required during the recording.

### 2.3 Paradigm

As illustrated in Figure 1, the experiment employed a roving paradigm presented within a block structure, consisting of 20 blocks separated by 35‐s silent intervals. Each block contained 150 trials with equal presentations of AM (220 Hz, 40 Hz) and AM (440 Hz, 80 Hz) tones, where each trial comprised a 450‐ms stimulus followed by a 550‐ms silent interstimulus interval. Within each block, the same AM tone was repeated 4–6 times consecutively before transitioning to the other tone type, with the number of repetitions pseudorandomly varied to minimize sequence regularity. In this roving structure, the first stimulus of each repetition train was designated as the deviant (except for the initial train of each block), all subsequent positions as nondeviant stimuli, with the final position specifically served as the standard.

To assess cortical change‐detection processes, each block included 30 transition pairs pseudorandomly interspersed throughout the sequence. These pairs consisted of 15 ascending transitions (AM (220 Hz, 40 Hz) followed by AM (440 Hz, 80 Hz)) and 15 descending transitions (AM (440 Hz, 80 Hz) followed by AM (220 Hz, 40 Hz)). For counterbalancing, the presentation order of the tones alternates between blocks, with odd‐numbered blocks starting with AM (220 Hz, 40 Hz) and even‐numbered blocks starting with AM (440 Hz, 80 Hz). The total experimental duration is approximately 61 min (20 blocks × 185 s per block).

### 2.4 EEG Recordings

We collected scalp EEG data using a Compumedics Neuroscan device with two recording Ag/AgCl sintered electrodes according to the 10/20 International System. The montage consisted of a ground electrode at the center of the forehead (Fpz), a reference electrode on the right earlobe (A2), and two recording electrodes at Cz and Fz positions. All electrodes were secured using Ten20 conductive paste and stabilized with medical tape, with impedances maintained below 10 KΩ. The recording electrodes were acquired through the SynAmps RT bipolar inputs, configured to share a common reference at A2. Data were digitized at a 20‐kHz sampling rate to capture the high‐frequency FFRs, with no online filters applied during acquisition. Stimulus onset markers were sent to the amplifier through a Cedrus StimTracker device.

### 2.5 Auditory Response Analysis

#### 2.5.1 Group‐Level Characteristics Analysis

##### 2.5.1.1 Sustained Components

We first characterized frequency‐tagged responses at the group level by comparing the power spectral density (PSD) difference between stimulus conditions and resting state (RS), isolating evoked activity and reducing the influence of nonspecific background activity. Three spectral components were analyzed: (1) low‐frequency ASSR (1–2 Hz) induced by the fixed 1‐s stimulus onset asynchrony (SOA), (2) gamma‐band ASSRs at modulation frequencies (40 and 80 Hz), and (3) high‐frequency FFRs at carrier frequencies (220 and 440 Hz).

For the 1‐Hz ASSR, we preprocessed the continuous EEG data by down‐sampling to 100 Hz and applying a band‐pass filter between 0.1 and 20 Hz. Each 150‐s experimental block was divided into 10 nonoverlapping 15‐s epochs (−0.1 to 15 s), followed by baseline correction using the 100‐ms prestimulus interval and averaging. RS periods were processed identically, with each 30‐s period divided into two 15‐s epochs and then averaged. The averaged signals were filtered using a second‐order Butterworth band‐pass filter (0.5–2.5 Hz) to isolate the frequency range of interest. PSD was computed using the discrete Fourier transform (DFT), with zero‐padding to achieve 0.0125‐Hz frequency resolution. To facilitate between‐subject comparisons, the power spectrum for each participant was normalized by dividing each frequency bin's power by the total power within the analyzed band.

Statistical significance was assessed with spatiospectral cluster‐based permutation testing (Maris and Oostenveld 2007) implemented in MNE‐Python, applied to normalized PSD values from Fz and Cz. For each frequency bin, we ran paired *t*‐tests across participants contrasting stimulus versus RS conditions and used threshold‐free cluster enhancement (TFCE) to form clusters on the sensor × frequency grid. Significance was evaluated with 2000 permutations (*α* = 0.05, one‐tailed) to test for frequency‐specific power increases during stimulus presentation. In the results, cluster onset and offset (Hz) are defined as the first and last frequency bins where both Fz and Cz were significant (sensor‐wise conjunction), and we report the corresponding peak statistic.

For gamma‐band ASSRs and high‐frequency FFRs, the continuous EEG data were band‐pass filtered (2–1500 Hz) and notch filtered at 60 Hz and its harmonics. The data were then segmented into epochs (−100 to 450 ms), with trials rejected using a ±150‐μV threshold. To avoid contamination from transition responses, only nondeviant stimuli (all positions except the first within each tone train) were included in the analysis. The accepted trials were then averaged separately for the two AM conditions, followed by band‐pass filtering using Butterworth filters optimized for each frequency component (Table 1). PSD was computed on the 0–450‐ms poststimulus window with 0.25‐Hz frequency resolution achieved through zero‐padding. Statistical analyses followed the same cluster‐based permutation procedures described above, including the same criteria for significant cluster identification.

**TABLE 1:** Filter parameters and trial counts for sustained component analysis.

Component	1‐Hz ASSR	40‐Hz ASSR	80‐Hz ASSR	220‐Hz FFR	440‐Hz FFR
Filter band/Hz	0.5–2.5	25–55	65–95	200–280	420–540
Filter order	2^nd^	2^nd^	2^nd^	4^th^	4^th^
Number of trial	200	1140 ± 44	1138 ± 40	1140 ± 44	1138 ± 40

##### 2.5.1.2 Transient Components

For ERP analyses, the continuous EEG data were down‐sampled to 250 Hz and band‐pass filtered (2–20 Hz). Subsequent preprocessing followed the same procedures as described for ASSRs and FFRs, including epoch segmentation (−100 to 900 ms), baseline correction, and artifact rejection (±150‐μV threshold). We analyzed four aspects of the transient neural responses: (1) the N1‐P2 complex, by separately comparing nondeviant responses with RS (AM (220 Hz, 40 Hz): 1180 ± 40; AM (440 Hz, 80 Hz): 1181 ± 31; RS: 1180 ± 35 accepted trials, mean ± SD across participants); (2) stimulus‐specific effects, by comparing responses between the two nondeviant AM conditions; (3) transition responses, by comparing deviant with standard responses in both ascending and descending transitions; and (4) directional asymmetry in transition responses, assessed by comparing difference waves (deviant—standard) between ascending and descending transitions, with both transition types containing approximately 295 ± 7–10 trials per AM condition.

Statistical inference also used spatiotemporal cluster‐based permutation testing across Fz and Cz: paired *t*‐tests were computed at each time sample across participants, TFCE was applied on the sensor × time grid, and significance was assessed with 2000 permutations (α = 0.05, two‐tailed). We report each significant cluster's onset/offset (ms) where Fz and Cz were both significant (sensor‐wise conjunction) and the peak test statistic.

#### 2.5.2 Individual‐Level Sensitivity Analysis

Given the distinct signal characteristics of sustained and transient neural responses, we employed different analytical approaches. For sustained components that require extensive trial averaging due to very low single‐trial amplitudes, we applied permutation testing on power spectra derived from individual‐averaged responses. In contrast, for transient components that maintain adequate SNR with block‐level averaging and comprise multiple overlapping components, we employed multivariate classification across independent blocks.

##### 2.5.2.1 Sustained Components

We implemented a frequency‐wise permutation analysis of the normalized PSD increase to assess individual sensitivity. Specifically, for each participant, we computed the PSD difference between stimulus conditions and RS, considering only positive differences, and normalized these values to their total power. Analyses focused on specific frequency bands of interest: the low‐frequency ASSR (1 Hz and its harmonic at 2 Hz, ±0.1 Hz around each center frequency), gamma‐band ASSRs (40 Hz and 80 Hz, ±2 Hz), and FFRs at 220 and 440 Hz with upper sidebands at 260 and 520 Hz (±2 Hz). Lower sidebands (180 and 360 Hz) for FFRs were excluded because they exactly coincide with 60‐Hz power line harmonics, rendering neural activity indistinguishable from electrical noise.

Statistical significance was determined using permutation testing, where each frequency bin was compared against the mean of all other bins. This approach controls for frequency specificity by identifying only genuine power elevations relative to the surrounding spectrum. The observed difference was compared against a null distribution generated from 2000 random permutations, with *p*‐values calculated as the proportion of permuted differences exceeding the observed value. A spectral component was considered present in an individual if at least three consecutive points within the fundamental frequency or its harmonic/sideband ranges showed *p*‐values < 0.05 at the Fz electrode.

##### 2.5.2.2 Transient Components

We implemented a machine‐learning (ML) approach that balanced signal reliability and sample size constraints by combining block‐wise averaged responses from both Fz and Cz electrodes. The analysis was performed on band‐pass filtered data (2–20 Hz) using epochs from −100 to 900 ms relative to stimulus onset, with a focus on the 0–900‐ms period containing the primary ERP components. For each participant and condition comparison, we constructed classification samples by averaging the 20 blocks separately for each electrode (Fz and Cz), yielding 40 classification samples per condition (20 blocks × 2 electrodes).

For each of the comparisons described above, the classification pipeline employed a linear support vector machine (SVM) implemented in scikit‐learn (Pedregosa et al. 2011) with the default regularization parameter (C = 1.0) and stratified 20‐fold cross‐validation. Data were standardized using the training set statistics within each fold to ensure robust scaling across features while preventing data leakage. Statistical significance was evaluated through a permutation framework (2000 iterations). Classification accuracy was computed using true labels and compared against a null distribution obtained from identically cross‐validated models trained on randomly permuted labels. Transient ERP components were considered reliably detected when the average classification accuracy exceeded the 95th percentile of the null distribution (*p* < 0.05).

#### 2.5.3 Recording‐Duration Analysis

To determine the minimum recording duration required for reliable detection of each auditory component, we systematically evaluated detection sensitivity as a function of cumulative data length. For each participant, component‐specific analyses were performed by incrementally adding blocks of data, starting from a single block (3 min) and continuing up to the full 20 blocks. Sensitivity curves were generated by calculating detection rates at each cumulative block increment.

For sustained components (FFRs and ASSRs), individual‐level detection was evaluated by applying the same permutation‐based spectral analysis described in Section 2.5.2 to cumulatively averaged responses at each increment. For transient components (N1–P2 and transition responses), the same ML classification framework (linear SVM with *n*‐fold cross‐validation, where *n* equaled the number of available blocks) was used, based on features from the 0–900‐ms poststimulus window at Fz and Cz electrodes. For the initial single‐block condition (3 min), a twofold cross‐validation procedure was applied to ensure unbiased performance estimation. Component detection criteria and significance thresholds were identical to those used in the individual‐level analyses. Stability was defined as the minimum number of blocks required for detection sensitivity (i.e., the proportion of participants with significant responses) to reach and maintain ≥ 0.90 across subsequent increments.

## 3 Results

### 3.1 Sustained Components

**FIGURE 2:**
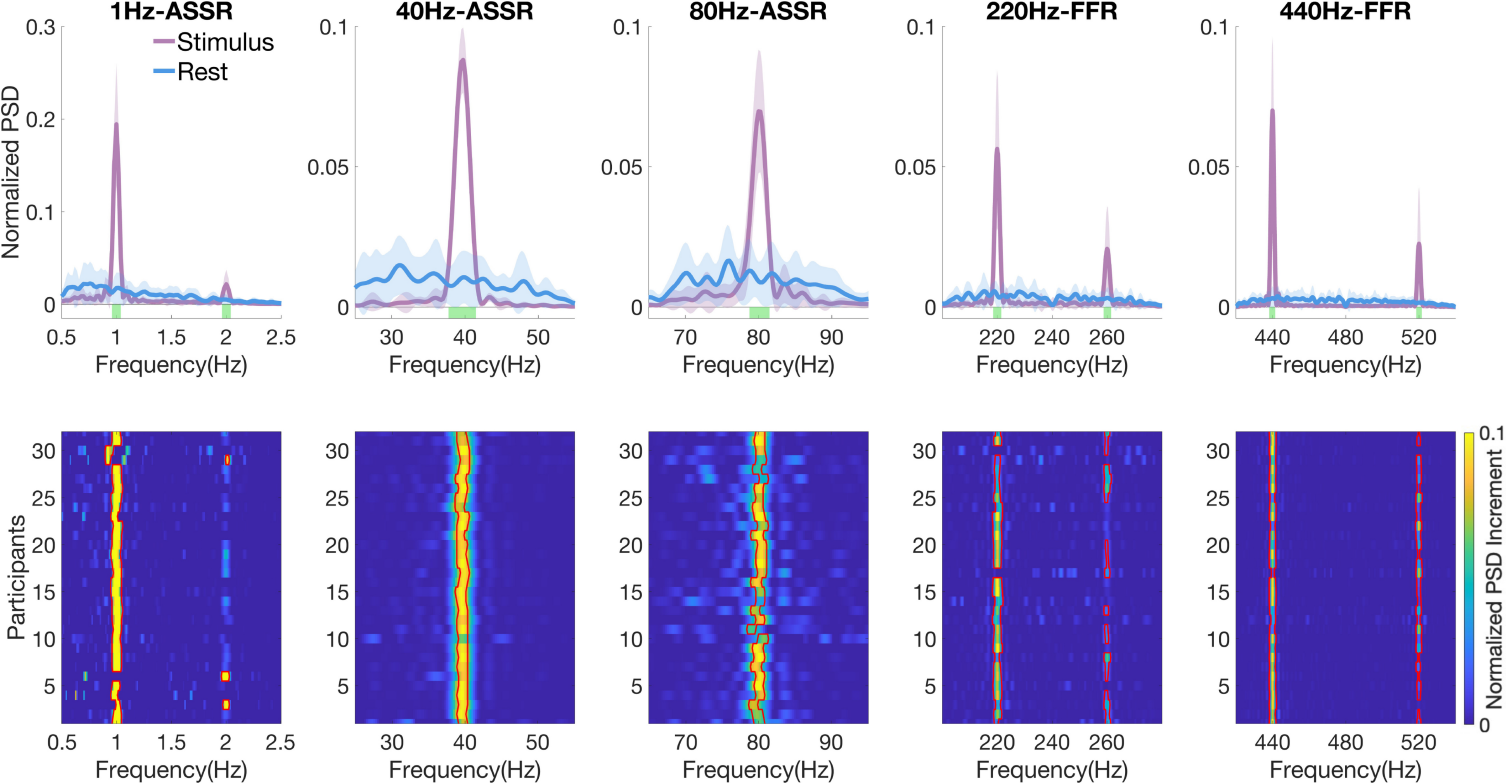
Spectral analysis of frequency‐tagged sustained components. Upper panels: Group‐level normalized PSD for stimulus (purple) and resting state (blue) at Fz across five sustained components; shaded areas indicate standard error across participants. Colored bars below each plot mark significant frequency clusters (cluster‐wise *p* < 0.05). Lower panels: Individual‐level maps of normalized PSD increment with permutation‐based significance; each row represents an individual participant, color denotes the magnitude of PSD increment, and red boxes outline significant frequency ranges. When considering either fundamental or harmonic/sideband responses, all components achieved 100% detection sensitivity across participants.

At the group‐level, cluster‐based permutation tests on normalized PSD between stimulus conditions and RS indicated significant spectral power increases in all targeted frequency ranges (Figure 2, upper panel). Specifically, the 1‐Hz ASSR exhibited prominent responses at both the stimulus presentation rate (cluster: 0.96–1.04 Hz, cluster‐wise *p* < 0.05) and its harmonic frequency (1.96–2.04 Hz, cluster‐wise *p* < 0.05), with within‐cluster peaks at 0.98 Hz (*t* = 6.75) and 2 Hz (*t* = 1.29), respectively. For gamma‐band ASSRs, significant clusters were observed around modulation frequencies (cluster: 37.75–41.50 Hz, cluster‐wise *p* < 0.05; and 78.75–81.50 Hz, cluster‐wise *p* < 0.05), with within‐cluster peaks at 39.75 Hz (*t* = 26.95) and 80.25 Hz (*t* = 7.27). In the FFR range, significant clusters encompassed the carrier frequencies (cluster: 218.50–221.50 Hz, cluster‐wise *p* < 0.05; 438.25–441.50 Hz, cluster‐wise *p* < 0.05) and their upper sidebands (cluster: 258.75–261.50 Hz, cluster‐wise *p* < 0.05; 518.50–521.50 Hz, cluster‐wise *p* < 0.05), with within‐cluster peaks at 220.50 Hz (*t* = 5.16), 259.75 Hz (*t* = 1.77), 440.00 Hz (*t* = 9.61), and 519.50 Hz (*t* = 1.84).

At the individual level (Figure 2, lower panel), we observed varying detection rates across frequency ranges. For low‐frequency ASSR, 31/32 participants showed a significant response at 1 Hz, whereas only three participants showed a detectable 2‐Hz harmonic. Notably, Participant #6 showed a significant response exclusively at the 2‐Hz harmonic without a detectable 1‐Hz component. For gamma‐band ASSRs, significant responses were present in all participants (32/32) at both 40 and 80 Hz. For FFRs, significant responses were detected in 30/32 participants at the 220‐Hz carrier and in all participants at the 440‐Hz carrier; the corresponding upper sidebands showed lower detection rates (19/32 at 260 Hz and 27/32 at 520 Hz). Interestingly, two participants (#17 and #30) showed significant responses only at the 260 Hz sideband without a detectable 220‐Hz carrier frequency response. Considering either the fundamental or harmonic/sideband responses, all sustained components achieved 100% detection sensitivity across participants.

### 3.2 Transient Components

#### 3.2.1 Obligatory Responses to Nondeviant Tones

**FIGURE 3:**
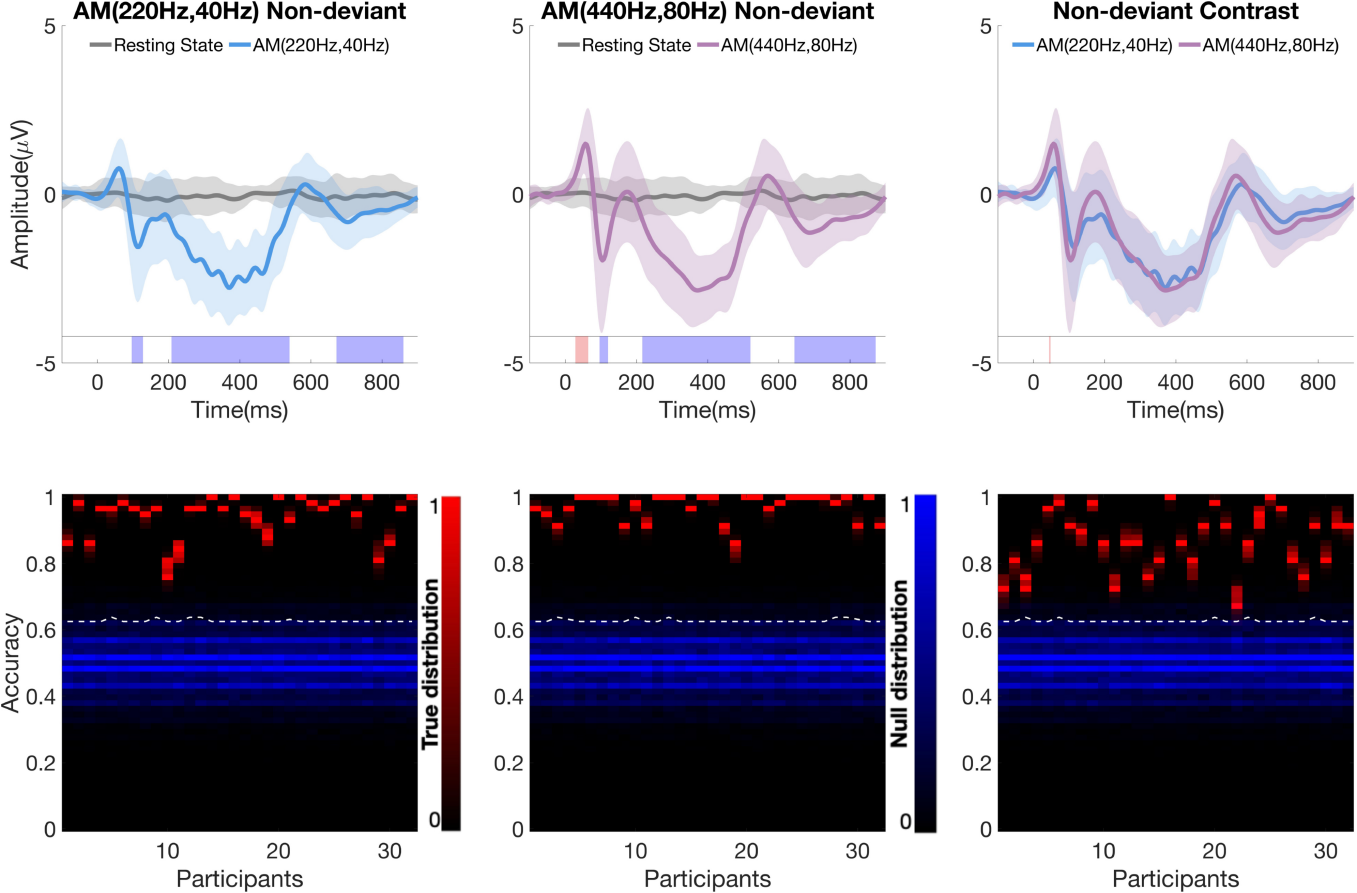
Obligatory responses to nondeviant AM tones. Upper panel: Grand‐averaged waveforms at Fz for AM (220 Hz, 40 Hz) vs. resting state (RS) (left), AM (440 Hz, 80 Hz) vs. RS (middle), and their contrast (right). Shaded bands indicate SEM across participants. Colored bars beneath the waveforms mark significant temporal clusters (cluster‐wise *p* < 0.05; red = positive, blue = negative). Lower panel: Individual‐level classification results showing accuracy distributions under true (red) and null (blue) label permutations for each participant (2000 permutations); white dashed lines indicate each participant's 95th‐percentile null threshold.

At the group‐level, cluster‐based permutation tests revealed significant temporal clusters for both AM tones compared with RS (Figure 3, upper panel). For AM (220 Hz, 40 Hz), three significant clusters were observed (cluster‐wise *p* < 0.05): a negative‐going cluster at 96–128 ms (within‐cluster peak *t* = 1.17 at 116 ms), falling in the latency range characteristic of the onset N1; a broad negative cluster spanning 208–540 ms (peak *t* = 15.06 at 420 ms) that covers much of the stimulation period, in a time window typical for sustained negativity to long tones; and a later negative cluster at 672–860 ms (peak *t* = 1.49 at 712 ms) occurring in the post‐offset interval. For AM (440 Hz, 80 Hz), four significant clusters were found (cluster‐wise *p* < 0.05): an early positive cluster at 28–64 ms (peak *t* = 2.46 at 52 ms), within the P1 window; a negative cluster at 96–120 ms (peak *t* = 1.02 at 108 ms), within the N1 window; a sustained negative cluster covering 216–520 ms (peak *t* = 195.60 at 424 ms) during the stimulus period; and a late negative cluster at 644–872 ms (peak *t* = 2.99 at 720 ms) after stimulus offset. Direct comparison between AM conditions revealed a stimulus‐specific effect in the P1 window (44–48 ms; cluster‐wise *p* < 0.05), with AM (440 Hz, 80 Hz) > AM (220 Hz, 40 Hz) (within‐cluster peak *t* = 0.72 at 48 ms).

At the individual level (Figure 3, lower panel), within‐subject classification confirmed robust obligatory responses. Stimulus versus RS decoding was significant for all participants for AM (220 Hz, 40 Hz) (accuracy 0.94 ± 0.06) and AM (440 Hz, 80 Hz) (0.96 ± 0.04), yielding 100% detection sensitivity (32/32). A direct discrimination between stimulus conditions was likewise significant for 32/32 participants (accuracy 0.86 ± 0.08), exceeding permutation‐based significance thresholds. Together, these results indicate that both nondeviant AM tones elicited a reliable onset N1, a sustained negative shift during the stimulus period, and a late post‐offset negativity, with stimulus‐specific differences most pronounced in the P1‐latency window.

#### 3.2.2 Change‐Detection Responses to Transitions

**FIGURE 4:**
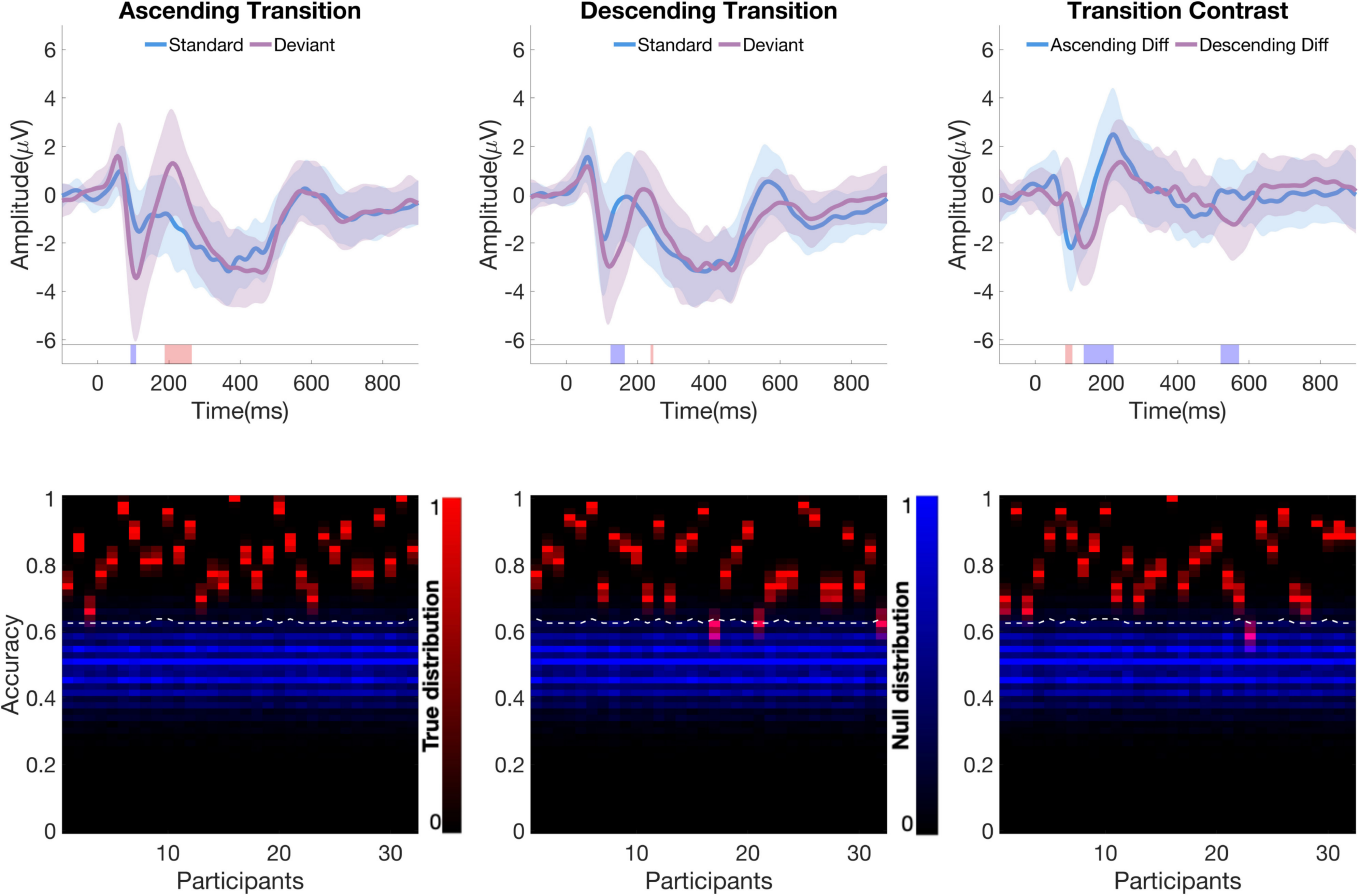
Change‐detection responses to AM tone transitions. Upper panel: Grand‐averaged waveforms at Fz for ascending (left) and descending (middle) transitions, showing standard (blue) and deviant (red) responses; the right panel shows the corresponding difference waves (deviant−standard). Shaded bands indicate SEM across participants. Colored bars beneath the waveforms mark significant temporal clusters (cluster‐wise *p* < 0.05; red = positive, blue = negative). Lower panel: Individual‐level classification results showing accuracy distributions under true (red) and null (blue) label permutations for each participant (2000 permutations); white dashed lines indicate each participant's 95th‐percentile null threshold.

At the group‐level, cluster‐based permutation tests (Figure 4, upper panel) identified significant temporal clusters for both ascending and descending transition conditions. For ascending, two clusters reached significance (cluster‐wise *p* < 0.05): a negative cluster at 92–108 ms (within‐cluster peak *t* = 0.71 at 100 ms; N1 window) and a positive cluster at 188–264 ms (peak *t* = 1.51 at 224 ms; P2 window). For descending, two clusters were significant (cluster‐wise *p* < 0.05): a negative cluster at 124–164 ms (peak *t* = 1.06 at 144 ms; MMN window) and a positive cluster at 236–244 ms (peak *t* = 0.67 at 240 ms; P3a window). A direct contrast (descending vs. ascending) revealed three significant clusters (cluster‐wise *p* < 0.05): 84–104 ms (peak *t* = 0.94 at 92 ms; early N1‐latency window), 136–220 ms (peak *t* = 1.24 at 168 ms; mid‐latency window spanning MMN/N1–P2 timing), and 520–572 ms (peak *t* = 0.90 at 560 ms; late window), indicating asymmetric temporal profiles for ascending versus descending transitions.

Single‐subject analysis further supported these differential patterns. For ascending transitions, all participants showed significant classification accuracy above chance (mean = 0.83 ± 0.09) with 100% detection sensitivity (32/32). In contrast, descending transitions yielded significant effects in 30/32 participants (mean = 0.81 ± 0.10; nonsignificant cases had accuracies of 0.60 ± 0.03 and 0.62 ± 0.03 with respective thresholds of 0.64). Between‐condition classification (ascending vs. descending transitions) achieved significance in 31/32 participants (mean = 0.82 ± 0.10), with participant #23 showing non‐significant accuracy of 0.59 ± 0.03 against a threshold of 0.63. These results align with the group‐level findings, with ascending transitions showing effects in the N1/P2 windows and descending transitions showing effects in the MMN/P3a windows.

### 3.3 Characterizing Response Stability Across Recording Duration

**FIGURE 5:**
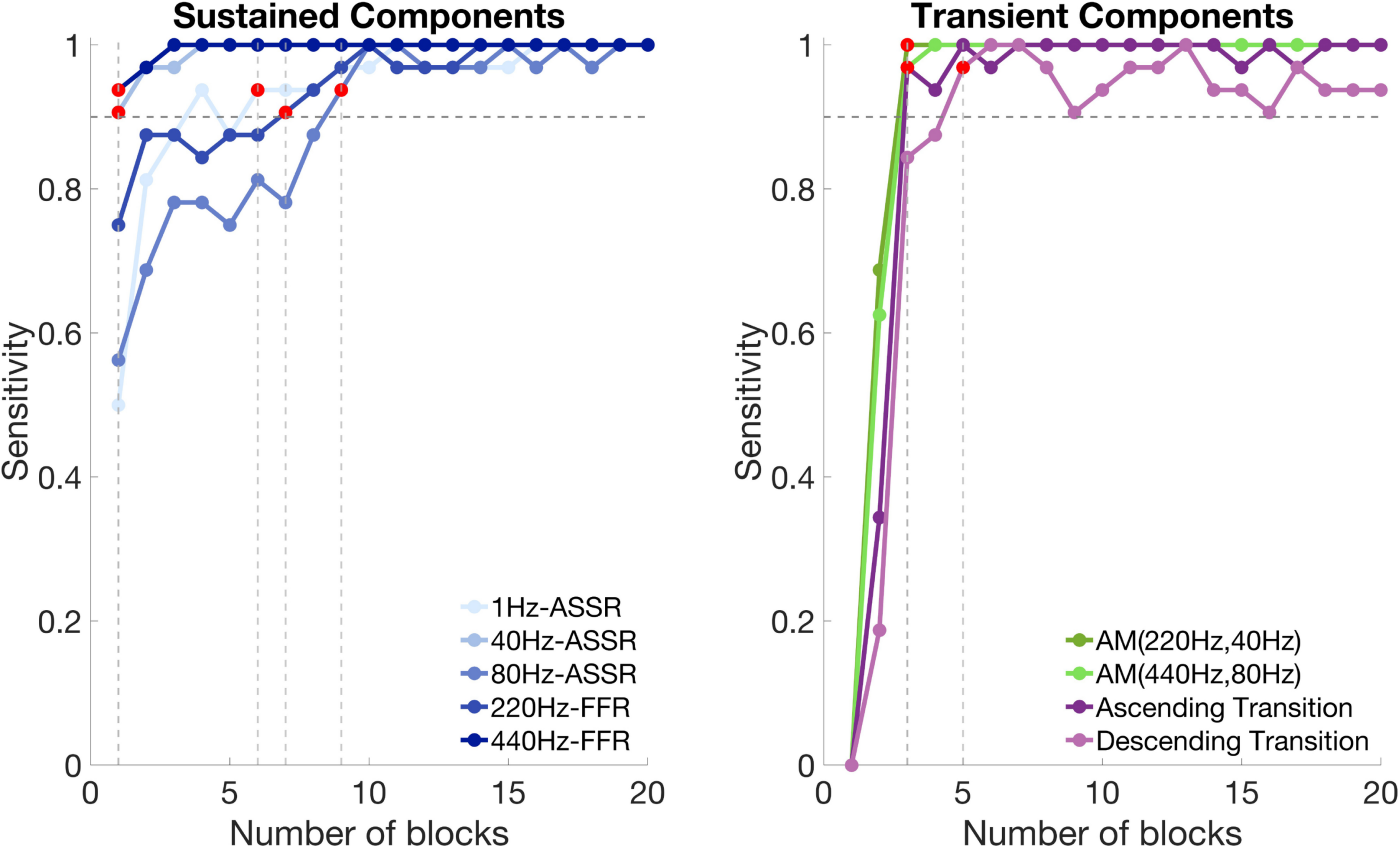
Stability of auditory response detection across cumulative recording duration. Left panel: Sensitivity curves for sustained components (ASSRs, FFRs) as a function of cumulative blocks. Right panel: Sensitivity curves for transient components, including obligatory responses to the two AM tones relative to RS and change‐detection responses to ascending and descending transitions. The dashed line marks the 0.90 detection‐sensitivity threshold. Red markers denote the earliest block at which sensitivity first reaches ≥ 0.90 and subsequently remains at or above this threshold.

Analysis of recording‐duration requirements revealed distinct stabilization thresholds across auditory components (Figure 5). Among sustained components, the 80‐Hz ASSR required the longest stabilization time (nine blocks and 27 min) to consistently achieve ≥ 0.90 detection sensitivity across participants. In contrast, the 40‐Hz ASSR stabilized within a single block (3 min). The 1‐Hz ASSR tracking the presentation rate reached stability after six blocks (18 min). For FFRs, we observed an inverse relationship between carrier frequency and required recording time: the 440‐Hz FFR stabilized after one block (3 min), whereas the 220‐Hz FFR required seven blocks (21 min) to achieve reliable detection.

For transient ERP components, the stimulus condition relative to RS reached ≥ 0.90 sensitivity after three blocks (9 min) for both AM tones, corresponding to an obligatory N1, a sustained negative shift and a post‐offset negativity. For transition responses, stability diverged by direction: ascending stabilized after three blocks (9 min), characterized by N1–P2 modulation; descending required five blocks (15 min) to reach stable detection, with effects in the MMN/P3a windows.

## 4 Discussion

This study introduced a frequency‐tagged roving paradigm designed to concurrently elicit multiple auditory‐evoked responses spanning subcortical to cortical levels, and evaluated its detection sensitivity in healthy individuals. The paradigm achieved 100% detection sensitivity for all sustained components, with phase‐locking observed in FFRs to carrier frequencies (220/440 Hz), ASSRs to modulation rates (40/80 Hz), and low‐frequency ASSR tracking the stimulus presentation rate (1 Hz). For transient ERP components, the obligatory N1 component and a sustained negative shift during stimulus presentation were detected in all participants in response to nondeviant stimuli, reflecting reliable cortical encoding of acoustic features. Furthermore, directional asymmetry was observed in transition responses: Ascending transitions predominantly elicited an N1–P2‐like complex (32/32 participants), while descending transitions elicited responses consistent with MMN and P3a in most participants (30/32 participants). All components reached ≥ 0.90 sensitivity within 27 min of recording. Collectively, these findings support the feasibility of using this paradigm for efficient multi‐level auditory response characterization in healthy individuals.

Regarding gamma‐band ASSRs specifically, the 100% detection rate across participants suggests the benefits of employing single AM tones within a roving design. In contrast, some earlier studies attempted to elicit multiple ASSRs simultaneously using multiplexed AM paradigms, wherein two or more modulation frequencies were applied to a single carrier (e.g., Slugocki et al. 2017). However, such designs may compromise frequency specificity. For example, Roß et al. (2000) demonstrated that simultaneous presentation of closely spaced modulation frequencies (e.g., 39 and 41 Hz) suppressed the 40‐Hz ASSR, indicating interference with phase‐locked neural responses. Similarly, Sonck et al. (2025) found that single‐modulation paradigms yielded significantly higher SNRs, supporting the view that concurrent modulations reduce response separability. Furthermore, the balanced trial structure of the roving design ensured that each AM condition was presented equally often in the nondeviant role. This enabled separate averaging of nondeviant trials to derive the 40‐ and 80‐Hz ASSRs, thereby supporting balanced SNR estimation for both components.

Our paradigm elicited FFRs at both 220 and 440 Hz, with clear phase‐locking observed in individual participants. These carrier frequencies were chosen based on evidence of local amplitude maxima at approximately 208 and 415 Hz, which are thought to result from constructive interference among multiple neural generators (Tichko and Skoe 2017). We observed consistent upper sideband responses at 260 and 520 Hz in 19/32 and 27/32 participants, respectively, which emerge as a direct consequence of the amplitude modulation (e.g., carrier + modulation frequencies: 220 + 40 Hz = 260 Hz). Notably, two participants (#17 and #30) showed significant responses exclusively at the 260‐Hz sideband without detectable 220‐Hz carrier FFR components. We speculate that this dissociation may result from the combined influence of individual encoding preferences (Coffey et al. 2019) and limited signal robustness. First, inspection of individual FFR spectra (Figure 2) revealed that while most participants showed stronger responses at the carrier frequency (220 Hz), a subset (including Participants #17 and #30) exhibited greater spectral power at the upper sideband (260 Hz). This pattern suggests that their carrier responses were relatively weaker (Ananthakrishnan et al. 2017), increasing the likelihood of falling below detection thresholds. Furthermore, suboptimal signal quality may render the already weak carrier response undetectable. For example, Participant #17 did not reach significance in the descending transition classification, potentially reflecting reduced signal robustness or other nonspecific variability. In our view, such individual‐level dissociations are consistent with normal intersubject variability and should not be considered abnormal in healthy participants.

Additionally, our study revealed a clear 1‐Hz stimulation‐rate entrainment response, matched to the presentation rate of the roving stimuli, with measurable peaks at the fundamental 1 Hz and its 2‐Hz harmonic. Unlike the gamma‐band ASSRs reflecting rapid phase‐locking to fine acoustic structure, this low‐frequency response likely reflects auditory‐cortical tracking of slower rhythmic patterns (Giraud and Poeppel 2012; Etard and Reichenbach 2019). Such slow temporal regularities are characteristic of natural acoustic phenomena such as phrase‐level units in speech and slow beat cycles in music. Taken together, these results demonstrate that the auditory system integrates information across temporal scales, from cortical entrainment at ~1 Hz to subcortical phase‐locking at 220 and 440 Hz, and that our paradigm provides a unified approach for concurrently assessing these distinct processing levels.

In response to nondeviant AM tones, our paradigm elicited a composite ERP pattern that enabled 100% classification of stimulus versus rest periods across all participants. Among these components, the N1 component, peaking near 100 ms, is consistent with canonical onset responses to sound and reflects obligatory cortical encoding of stimulus onset (Näätänen and Picton 1987; Picton et al. 2000). Subsequently, our data revealed a sustained negative deflection spanning ~200–500 ms during AM stimulation, an effect commonly observed with modulated or long‐duration tones and thought to reflect extended cortical tracking of temporal regularities (Picton et al. 2003). In the poststimulus period, we observed a post‐offset negative potential (~650–850 ms), peaking around 720 ms (~270‐ms post‐offset), consistent with an offset N2‐like component possibly reflecting late‐stage cortical processing of stimulus termination (Baltzell and Billings 2014). Given the consistent elicitation of these ERP components across participants, we attribute the observed detection rates to two key features of the paradigm. First, the block‐based stimulus structure enhanced SNR through within‐block averaging, while preserving a sufficient number of block‐level samples for classification. Second, interleaved rest periods allowed clear temporal segmentation of evoked activity from background fluctuations, supporting reliable detection of the distinct cortical responses described above.

Furthermore, our paradigm revealed direction‐specific neural dynamics in response to spectral transitions. Descending transitions elicited the expected MMN responses (124–164 ms), consistent with predictive error signaling in automatic deviance detection (Näätänen et al. 2019). Notably, the MMN cluster appears relatively brief compared to typical durations, likely due to the inherently conservative nature of cluster‐based permutation testing (Sassenhagen and Draschkow 2019), further constrained by our dual‐electrode significance criterion. By contrast, ascending transitions selectively enhanced an N1–P2‐like complex (88–112 ms and 192–260 ms), revealing an unanticipated asymmetry relative to the MMN responses observed for descending transitions. A similar phenomenon, an unexpectedly early negativity (~105 ms) to an ascending frequency deviant, was reported by Rutiku et al. (2024), who used a local–global paradigm in which the local effect was elicited by quintuple‐tone sequences (AAAAB) designed to evoke MMN. Structurally, brief segments within our roving design closely resemble the quintuple‐tone sequences, as both feature ascending frequency transitions embedded within short tone sequences composed of repeated tones.

Given this resemblance, we speculate two possible interpretations of the N1–P2‐like response observed in our paradigm. One possibility is that this early negativity reflects a temporally shifted MMN. For example, Peter et al. (2010) reported that ascending frequency deviants tend to elicit earlier and larger MMN responses than descending ones. This further suggests that ascending deviants may have been more perceptually discriminable, even though the magnitude of frequency change was matched for both transitions. Such heightened salience could have triggered involuntary attentional engagement (Näätänen 1990), thereby eliciting N1–P2 components instead of a classical MMN. Together, these results show direction‐specific differences in auditory change processing and demonstrate the sensitivity of our paradigm in capturing nuanced neural responses.

Our systematic analysis of recording duration revealed distinct stability thresholds across auditory components, reflecting differences in neural dynamics and providing empirical guidance for protocol optimization. For ASSRs, the 40‐Hz response stabilized rapidly (one block, 3 min), consistent with thalamocortical gamma‐band resonance enhancing phase‐locking (Bartos et al. 2007). In contrast, the 80‐Hz ASSR required nine blocks to stabilize, possibly reflecting a need for greater signal averaging to compensate for weaker neural synchronization and lower cortical SNR at higher modulation rates (Bidelman and Horn 2025). Interestingly, FFRs showed an inverse trend: the 440‐Hz response stabilized immediately (one block), whereas the 220‐Hz FFR required seven blocks. We speculate that this may be explained by superior constructive phase interference at 440 Hz, where aligned neural delays across brainstem generators maximize response coherence (Tichko and Skoe 2017). Notably, the 1‐Hz ASSR required six blocks to stabilize, which can be explained by its slow‐wave tracking that involves extensive distributed thalamocortical networks (Picton et al. 2003).

For transient responses to nondeviant AM tones, the composite ERP pattern, including the N1 onset response, sustained negativity, and post‐offset potential, stabilized rapidly within three blocks (9 min). Similarly, the N1–P2‐like activity evoked by ascending transitions also stabilized within three blocks. In contrast, the MMN elicited by descending transitions required five blocks to stabilize, possibly reflecting extended neural integration needed for automatic deviance detection, which relies on the comparison of current auditory input against sensory memory traces and prediction models built over longer timescales (Näätänen et al. 2007). Together, these results underscore that auditory response stabilization depends on component‐specific neural synchrony and generator dynamics. Although the full 61‐min protocol was used to empirically optimize detection thresholds, 0.91 individual‐level sensitivity was achieved within 27 min. This finding offers a practical reference point for efficient recording in resource‐limited or time‐constrained clinical settings, while recognizing the need for validation in patient populations.

Notably, there are two limitations in the present study. First, our minimal two‐channel EEG setup (Fz and Cz), though suitable for bedside use, limits our ability to characterize the neural generators of the observed ERP components. In particular, the present design does not permit source localization or topographic analysis, constraining the interpretation of phenomena such as the direction‐specific asymmetry in transition responses (e.g., MMN vs. N1–P2) and the differential stabilization profiles across components. High‐density EEG or MEG studies with source reconstruction would be needed to confirm whether distinct cortical networks or hierarchical processing stages are differentially engaged by ascending versus descending transitions.

Second, while the present study demonstrated high detection sensitivity and robustness in healthy participants, its clinical generalizability, particularly to DoC patients, remains to be validated. Critically ill patients differ from healthy individuals in several ways, including lower arousal levels, impaired sensory integration, and limited voluntary attention. Moreover, our classification strategy, which contrasts stimulus‐evoked activity against resting‐state EEG, assumes a relatively stable baseline, a condition that may not hold in patients, where pathological or highly variable resting activity could confound interpretation. Although the present design (e.g., block‐based stimulation, passive engagement, and short duration) was optimized for bedside application, its interpretability in patients with altered baseline dynamics requires empirical validation. Future studies should assess whether stimulus‐evoked responses and classification accuracy persist in patients with impaired consciousness states.

## 5 Conclusion

As demonstrated in this study, our frequency‐tagged roving paradigm elicited multiple auditory responses with high individual‐level sensitivity, enabling broad‐spectrum assessment of auditory processing across brainstem and cortical levels within a single session. These results lay important groundwork for future clinical validation, particularly in populations such as DOC, where rapid and passive assessment is essential. Notably, the paradigm achieved over 0.90 sensitivity for all components within 27 min using only two electrodes, indicating its potential utility in time‐constrained and resource‐limited clinical environments.

## Author Contributions

**Xiaoyu Wang:** conceptualization, methodology, software, validation, formal analysis, investigation, resources, data curation, writing – original draft, visualization. **Loretta Norton:** conceptualization, supervision, methodology, investigation, writing – review and editing. **Teneille E. Gofton:** supervision, investigation, writing – review and editing. **Derek B. Debicki:** supervision, investigation, writing – review and editing. **Marat Slessarev:** supervision, investigation, writing – review and editing. **Adrian M. Owen:** conceptualization, supervision, writing – original draft, writing – review and editing, funding acquisition.

## Funding

This work was supported by the Canadian Institute of Health Research Foundation (Grant 408004).

## Conflicts of Interest

The authors declare no conflicts of interest.

## Data Availability

The data supporting the findings of this research are available on request to the corresponding author, pending a formal data‐sharing agreement and approval from the local ethics committee.
